# Exploring sport friendship quality profiles in football participation among Chinese adolescents: a latent profile analysis

**DOI:** 10.3389/fpsyg.2026.1792610

**Published:** 2026-04-24

**Authors:** Qinghua Wu, Ying Zhao, Jieyou Zhou

**Affiliations:** 1 School of Basic Education, Guangdong Finance & Trade Vocational College, Qingyuan, China; 2 School of Sports Business, Guangzhou Sport University, Guangzhou, China; 3 School of Physical Education and Health Care, Sanming University, Sanming, China

**Keywords:** adolescent, football participation, latent profile analysis, sport friendship quality, sport friendship quality scale

## Abstract

Football benefits adolescents’ physical and mental health, but participation rates in China remain low despite government efforts. Few scholars have studied peer relationship differences as potential influences on football participation. This study investigated how sport friendship quality profiles relate to football participation and gender among adolescents. We recruited 450 adolescents (*M*_age_ = 12.46 years, *SD* = 1.78; range = 8–16 years; male = 214, female = 236) and used the Sport Friendship Quality Scale (SFQS) to measure six dimensions of peer relationships. Latent Profile Analysis (LPA) was employed to identify subgroups based on friendship quality. The BCH method was then used to test differences in football participation across profiles, and ANOVA was used to examine gender differences. LPA revealed three distinct friendship quality profiles: a High-Need Group (16.6%) with low scores across all dimensions, and two other profiles characterized by consistently high scores (53.8%) and moderate but uneven scores (29.6%). Football participation differed significantly across profiles (χ^2^ = 17.22, *df* = 2, *p* < 0.001), with the High-Need Group showing the lowest engagement, followed by the moderate-score group, and the high-score group showing the highest level of participation. Friendship quality profiles and gender are associated with adolescent football participation. Findings highlight the need for targeted social interventions, particularly for the High-Need Group, and gender-specific strategies to support female engagement.

## Introduction

Football plays a vital role in promoting physical and mental health among adolescents (Eberl et al., 2019; Krustrup and Parnell, 2019). Regular participation in football has been associated with improved cardiovascular fitness, muscular strength, and coordination (Krustrup et al., 2010), as well as enhanced psychological well-being and social development (Oja et al., 2015). As a team sport, football provides opportunities for physical exercise while also teaching important social skills (Friedrich and Mason, 2017). The nature of football requires players to communicate, cooperate, and develop strategies together, which can enhance interpersonal abilities. The psychological benefits are equally important, as being part of a team can boost self-esteem and provide a sense of belonging (Eberl et al., 2019). The structured environment of sports offers adolescents a space to experience both success and failure in a supportive context, building resilience and helping young people develop coping skills that transfer to other areas of life.

Recognizing these benefits, the Chinese government has actively promoted youth football through nationwide school programs and policy initiatives over the past decade (Peng et al., 2023). Despite these efforts, however, adolescent football participation rates in China remain lower than expected (Qian et al., 2017). This pattern is not unique to China; participation in youth sports has been declining in many countries (Zeng and He, 2024). Studies suggest this decline may be related to changing lifestyles and increased academic pressures (Birgersson et al., 2024; Chaput et al., 2020). Many adolescents today spend more time on sedentary activities and less time engaged in physical play compared to previous generations (Chapman et al., 2024).

Understanding the factors that influence football participation is therefore crucial for promoting youth engagement. While practical considerations like access to facilities and coaching are important, the social aspects of sports participation deserve particular attention (Bokhorst et al., 2010; Lin et al., 2024). One potentially important but understudied factor is the quality of peer relationships within sports settings. This connection between friendship quality and sports participation warrants careful examination. Different patterns of peer relationships may have distinct effects on adolescents’ engagement and commitment to football. By identifying these relationship patterns, we can better understand how to create supportive environments that encourage sustained participation.

High-quality friendships in sports settings play a crucial role in shaping adolescents’ athletic experiences and outcomes (Weiss and Smith, 2002). Friendship quality refers to the positive and negative features of close relationships between peers. Researchers measure it to understand how friendships influence an individual’s emotions, motivation, and behavior (Weiss et al., 1996; Weiss and Smith, 1999). The Sport Friendship Quality Scale (SFQS) identifies six key aspects: self-esteem support (e.g., encouragement), loyalty and trust (e.g., keeping secrets), shared interests (e.g., similar values), enjoyable time together (e.g., having fun), conflict management (e.g., solving arguments), and negative interactions (e.g., fights). These dimensions help explain why some sports friendships boost confidence and enjoyment while others create stress.

Research consistently demonstrates that positive peer relationships in sports contribute to enhanced motivation, increased enjoyment, and greater persistence in physical activities (Moran and Weiss, 2006; Weiss and Smith, 2002). These friendships provide emotional support during challenging moments, create shared memories through collective experiences, and foster a sense of belonging to a social group (Simpkins et al., 2013). Adolescents with strong sport friendships report higher levels of self-esteem and perceived competence, which are key correlates of continued participation. The reciprocal nature of these relationships means that better friendships lead to more engagement, which in turn strengthens the friendships, creating a positive cycle of participation and social connection (Moran and Weiss, 2006). Moreover, sport friendships often serve as protective factors against stress and anxiety, helping adolescents cope with competitive pressures.

The benefits of friendship quality are particularly pronounced in football due to its inherently collaborative nature (Adams and Carr, 2019). As a team sport requiring constant communication and coordination, football creates unique opportunities for developing deep, meaningful peer relationships (Liu et al., 2023). Adolescents with strong team friendships demonstrate greater tactical understanding, better on-field decision-making, and improved conflict resolution skills. The shared experiences of training, competing, and traveling together foster bonds that extend beyond the pitch. These relationships provide emotional security that allows players to take risks, try new skills, and recover more quickly from mistakes. Importantly, the social support system within a football team can buffer against negative experiences like performance slumps or injuries (Petrie et al., 2014).

Most existing research on sport friendship quality has adopted variable-centered approaches, examining how individual dimensions relate to outcomes such as motivation or participation (Moran and Weiss, 2006; Weiss and Smith, 2002). While valuable, this approach assumes that the relationships among friendship dimensions operate similarly for all adolescents and cannot identify subgroups who experience peer relationships in qualitatively different ways. Person-centered approaches, such as latent profile analysis (LPA), address this limitation by identifying subgroups of individuals who share similar patterns across multiple dimensions simultaneously (Howard and Hoffman, 2018). Rather than treating friendship dimensions as separate predictors, LPA examines how they combine naturally within individuals, revealing distinct profiles that may relate differently to outcomes. This approach has been successfully applied in sport psychology to identify motivation profiles (Lindwall et al., 2017; Zhao et al., 2025) and physical literacy patterns (Brown et al., 2020). Applying LPA to sport friendship quality could reveal why some adolescents thrive in football while others discontinue participation, helping coaches create more supportive team environments.

Traditional research often examines friendship quality by looking at single factors in isolation, which may ignore the finer-grained detail (Howard and Hoffman, 2018). Person-centered approaches like LPA offer a better way to understand how multiple friendship aspects combine naturally among adolescents. LPA identifies groups of individuals with similar patterns across various friendship dimensions, such as trust, conflict, and companionship. This method acknowledges that friendships work as whole systems, not just separate parts. Unlike traditional methods that assume all athletes experience friendships the same way, LPA reveals meaningful subgroups that may respond differently to coaching or team environments.

Latent profile analysis has demonstrated strong applicability in sports research, effectively identifying meaningful patterns in youth sports participation and social relationships (Brown et al., 2020; Lindwall et al., 2017). For example, researchers have used latent profile analysis to identify distinct motivation profiles in youth sports participation, which helps develop targeted interventions for behavioral weight loss programs (Hagerman et al., 2023). In addition, recent research has employed latent profile analysis to categorize adolescent personality types, which helps inform targeted interventions to improve physical activity levels (Chen et al., 2024).

Football participation naturally involves frequent social interaction, making peer relationships particularly significant. Recognizing these different friendship patterns helps explain why some teenagers stay engaged while others drop out. Coaches and program leaders can use these insights to develop specific activities that strengthen team relationships. This approach enables adults to create inclusive environments where all participants can grow both socially and athletically.

This study identifies distinct sport friendship quality profiles among Chinese adolescent football participants using LPA. We hypothesize that: (a) meaningful subgroups exist based on friendship quality dimensions, and (b) football participation levels differ significantly across these latent profiles. The findings will inform targeted interventions to enhance peer relationships and promote sustained engagement.

## Methods

### Participants

This research recruited 538 participants from Guangzhou through convenience sampling between April 1 and May 30, 2025. After excluding invalid responses (e.g., blank or patterned responses), our final sample included 450 adolescents (*M* age = 12.46 years, *SD* = 1.78; range = 8–16 years; male = 214, female = 236). This sample size meets recommended requirements for structural equation modeling and latent profile analysis (Spurk et al., 2020; Tein et al., 2013).

### Measures

#### Sport friendship quality

We assessed sport friendship quality using the Chinese version of the Sport Friendship Quality Scale (SFQS), originally developed by Weiss and Smith (1999) and subsequently adapted and validated for Chinese adolescents by Zhu and colleagues (Zhu et al., 2010). This scale assesses six dimensions of peer relationships in sports: connections and collaborative support (9 items, *α* = 0.862), conflict resolution (3 items, *α* = 0.742), positive character traits (3 items, *α* = 0.818), companionship and pleasant play (4 items, *α* = 0.816), self-esteem enhancement (3 items, α = 0.862), and sport supportiveness (3 items, *α* = 0.861). The scale comprises 25 items in total, with responses ranging from 1 (not at all true) to 5 (very true). Previous studies have demonstrated strong reliability and validity for the SFQS in youth sports contexts (Smith et al., 2006; Ullrich-French and Smith, 2006). In this study, the internal consistency (as shown in Table 1) and validity [χ^2^ (260) = 907.653, RMSEA = 0.074, CFI = 0.900, TLI = 0.885, SRMR = 0.046] are acceptable.

**Table 1:** Results of descriptive statistics and correlations.

	Variables	*M*	*SD*	1	2	3	4	5	6	7	8
1	Connections and collaborative support	4.023	0.683								
2	Conflict resolution	4.102	0.818	0.609^***^							
3	Positive character traits	4.191	0.841	0.708^***^	0.596^***^						
4	Companionship and pleasant play	4.056	0.838	0.683^***^	0.574^***^	0.608^***^					
5	Self-esteem enhancement	4.160	0.925	0.704^***^	0.585^***^	0.714^***^	0.691^***^				
6	Sport supportiveness	3.924	1.001	0.643^***^	0.516^***^	0.594^***^	0.692^***^	0.773^***^			
7	Football engagement	−0.094	0.702	0.195^***^	0.086	0.150^***^	0.113^*^	0.130^**^	0.181^***^		
8	Gender	/	/	0.039	0.042	0.086	0.053	0.080	0.013	−0.125^***^	
9	Age	12.460	1.778	0.048	0.062	0.120^*^	−0.024	0.066	0.035	0.209^***^	0.010

^*^p < 0.05, ^**^p < 0.01, ^***^p < 0.001.

#### Football participation

We measured football participation through participant self-reports of their engagement during the previous 7 days, using two specific questions: (1) “Besides school-arranged football activities, how many days did you participate in football activities in the past 7 days?” and (2) “Besides school-arranged football activities, how long did you participate in football activities each time in the past 7 days?” Total weekly minutes were calculated by multiplying days by duration per session. These total minutes were then standardized into z-scores. Following established statistical practices, we identified and removed extreme outliers with standardized scores below −3.29 or above +3.29 to ensure data quality and meet the assumptions of parametric tests.

### Procedure

The research team obtained ethical approval from the university’s Institutional Review Board (IRB: 2025LCLL-51) and secured written informed consent from all participating students, their parents, and school administrators from April to May, 2025. Teachers supervised participants as they completed paper-based questionnaires in classroom settings, with most students finishing in 10–15 min. To acknowledge their contribution, we provided all respondents with small appreciation gifts after they completed the survey.

### Data analysis

We analyzed the data using SPSS 25.0 for descriptive statistics, correlations, and ANOVA, and employed Mplus 8.3 for LPA of the SFQS dimensions. Following established methodological guidelines (Ferguson et al., 2020), we evaluated models with 1 to 6 profiles to identify the optimal solution. We assessed model fit using multiple criteria: lower values of AIC, BIC, and aBIC indicated better fit, while significant *p*-values for BLRT and LMR tests showed improvement over simpler models. Additionally, entropy values of 0.8 or higher confirmed accurate profile classification (Tein et al., 2013).

To examine differences in football participation across the latent profiles while accounting for classification uncertainty, we employed the three-step BCH method (Asparouhov and Muthén, 2014). After establishing the unconditional LPA model, we saved the posterior probabilities of profile membership. We then used the AUXILIARY BCH option in Mplus to test for differences in the distal outcome (football participation) across the latent profiles. This approach provides adjusted means and standard errors for the outcome within each profile and performs pairwise comparisons using Wald chi-square tests, all while correcting for the probability of profile misclassification. This method is superior to traditional two-step approaches that assign individuals to their most likely profile, as it prevents bias from classification error. To test whether gender predicted profile membership, we included gender as a covariate in the LPA model using the three-step method (R3STEP in Mplus).

## Results

### Multicollinearity

Multicollinearity occurs when two or more independent variables in a regression model are highly correlated, making it difficult to determine their individual effects on the dependent variable (Alin, 2010; Kim, 2019). A common measure to detect multicollinearity is the Variance Inflation Factor (VIF), where a VIF value above 10 indicates severe multicollinearity, while values between 5 and 10 suggest moderate concerns. Another indicator is tolerance, calculated as 1/VIF, with values below 0.2 raising potential issues. High multicollinearity inflates standard errors, reduces statistical significance, and may lead to unreliable coefficient estimates.

Table 2 presents the tolerance and VIF values for the six dimensions of the SFQS. All VIF values fall below 4 (range: 1.847–3.564), and tolerance values exceed 0.2 (range: 0.281–0.542), indicating no severe multicollinearity among the variables. The highest VIF (3.564) corresponds to Self-Esteem Enhancement, while the lowest (1.847) belongs to Conflict Resolution.

**Table 2:** Results of VIF.

	Variables	Tolerance	VIF
1	Connections and collaborative support	0.358	2.793
2	Conflict resolution	0.542	1.847
3	Positive character traits	0.389	2.569
4	Companionship and pleasant play	0.394	2.541
5	Self-esteem enhancement	0.281	3.564
6	Sport supportiveness	0.348	2.877

### Descriptive statistics

Table 1 displays the means, standard deviations, and correlations among the six SFQS dimensions, football engagement, gender, and age. The mean scores for SFQS dimensions ranged from 3.924 (Sport Supportiveness) to 4.191 (Positive Character Traits), indicating generally high perceived friendship quality. Football engagement showed a mean near zero (*M* = −0.094, *SD* = 0.702), reflecting standardized scoring. Correlation analyses revealed strong positive associations (*r* = 0.516–0.773, *p* < 0.05) among SFQS dimensions, with Self-Esteem Enhancement and Sport Supportiveness showing the strongest link (*r* = 0.773, *p* < 0.001). Football engagement correlated weakly but significantly with most SFQS dimensions (*r* = 0.113–0.195, *p* < 0.05), except Conflict Resolution (*r* = 0.086, *p* = 0.07).

### Latent profile analysis

**Figure 1:**
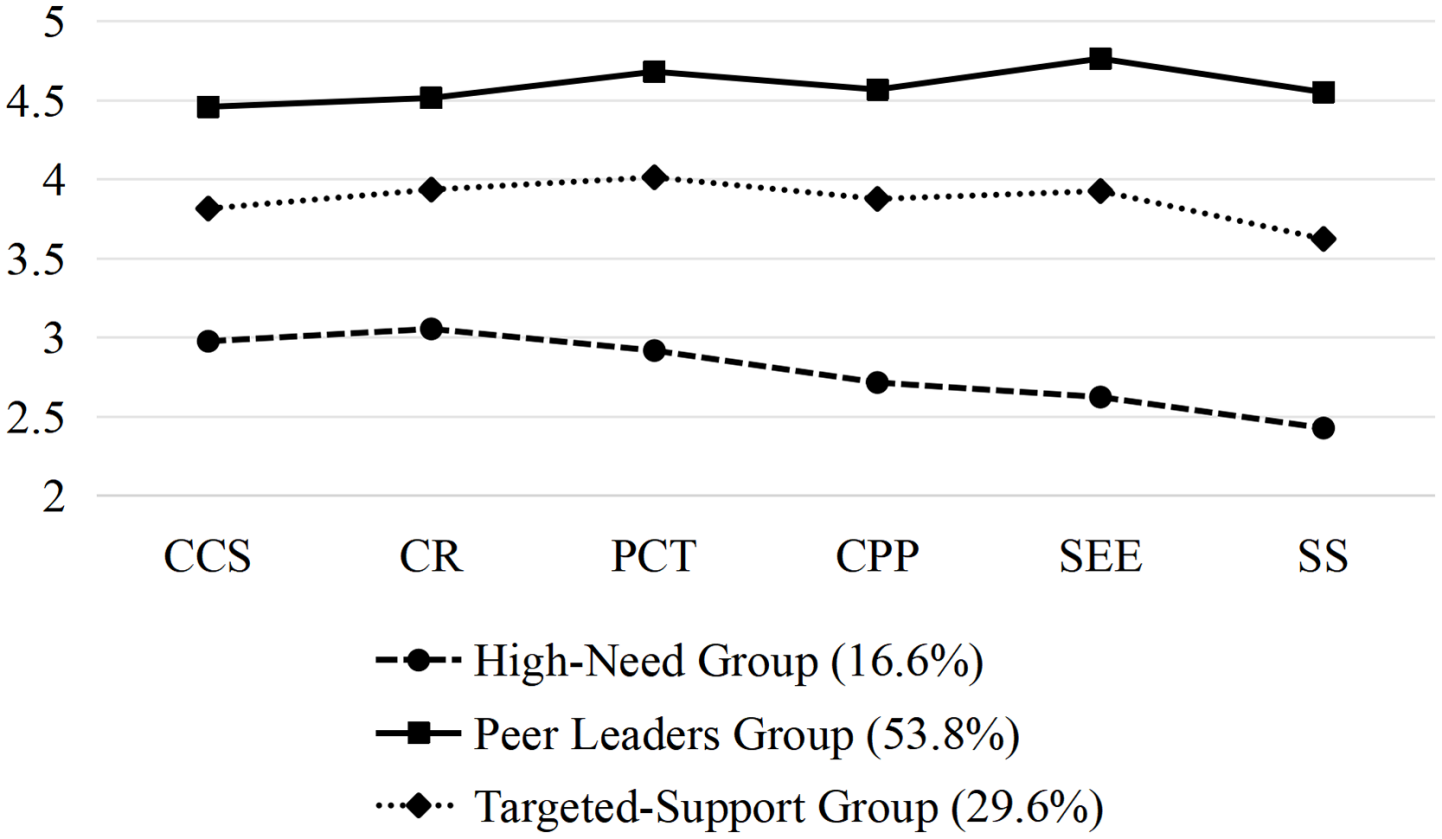
Friendship quality profiles of the best fitting model (three profiles). CCS = connections and collaborative support, CR = conflict resolution, PCT = positive character traits, CPP = companionship and pleasant play, SEE = self-esteem enhancement, SS = sport supportiveness.

We compared models with 1 to 6 latent profiles to identify the optimal solution for sport friendship quality patterns (Table 3). The fit indices showed consistent improvement as profiles increased: AIC decreased from 6766.98 (1-profile) to 4776.90 (6-profile), with similar trends for BIC and aBIC. Both BLRT and LMR tests indicated significant enhancements (*p* < 0.05) up to the 4-profile model, after which improvements plateaued (LMR *p* = 0.400 for 5-profile). Entropy values remained high (≥ 0.881) across models, with the 6-profile solution reaching 0.910. However, the 4-, 5-, and 6-profile models produced small subgroups (< 5% of sample), suggesting over-extraction. The 3-profile solution emerged as most appropriate, demonstrating excellent classification accuracy (entropy = 0.881) and comprising three substantial subgroups (Figure 1).

**Table 3:** Latent profile model information criteria, likelihood ratio test, and entropy (*n* = 450).

Model	*K*	Log (*L*)	AIC	BIC	aBIC	Entropy	BLRT	LMR	Group size
1	12	−3371.489	6766.979	6816.289	6778.206	/	/	/	/
2	19	−2662.198	5362.396	5440.472	5380.173	0.961	<0.001	<0.001	0.220/0.780
3	26	−2495.789	5043.577	5150.418	5067.904	0.881	<0.001	0.004	0.166/0.538/0.296
4	33	−2403.054	4872.107	5007.712	4902.983	0.894	<0.001	<0.001	0.147/0.507/0.049/0.296
5	40	−2370.974	4821.949	4986.319	4859.374	0.899	<0.001	0.400	0.063/0.133/0.035/0.269/0.501
6	47	−2341.449	4776.898	4970.033	4820.873	0.910	<0.001	0.411	0.050/0.125/0.021/0.036/0.272/0.496

Specifically, Profile 1 (16.6%, *n* = 74) demonstrated the lowest scores across all friendship quality dimensions, with particularly weak performance in Sport Supportiveness (*M* = 2.43) and Self-Esteem Enhancement (*M* = 2.62). These adolescents showed limited abilities in both collaborative support (*M* = 2.98) and conflict resolution (*M* = 3.05), suggesting they represent a group of struggling social learners who may benefit from targeted social skill development interventions, warranting the label “High-Need Group.”

Profile 2 (53.8%, *n* = 243) exhibited consistently high scores on all dimensions (*M* > 4.45), with especially strong performance in Positive Character Traits (*M* = 4.68) and Self-Esteem Enhancement (*M* = 4.76). Their balanced strengths across companionship (*M* = 4.57), conflict resolution (*M* = 4.52) and other areas indicate they are well-rounded team players who likely serve as positive peer models in sports settings, earning the designation “Peer Leaders Group”.

Profile 3 (29.6%, *n* = 133) displayed moderate scores (3.62–4.01) with relative strengths in Conflict Resolution (*M* = 3.94) and Positive Character Traits (*M* = 4.01). While outperforming Profile 1, their lower Sport Supportiveness (*M* = 3.62) suggests these emerging connectors have uneven social development and may need focused support to strengthen specific friendship quality aspects, justifying its classification as the “Targeted-Support Group.”

### Difference of football participation on potential subgroups

To examine differences in football participation across the latent profiles while accounting for classification uncertainty, we employed the BCH method (Asparouhov and Muthén, 2014). Results revealed significant overall differences across the three profiles (Wald χ^2^ = 17.22, *df* = 2, *p* < 0.001).

Pairwise comparisons using Wald tests indicated that the Peer Leaders Group (Profile 2, *M* = 0.01, *SE* = 0.05) reported significantly higher football participation than the Targeted-Support Group (Profile 3, *M* = −0.14, *SE* = 0.05, χ^2^ = 4.44, *df* = 1, *p* = 0.035). The Peer Leaders Group also demonstrated significantly higher participation than the High-Need Group (Profile 1, *M* = −0.33, *SE* = 0.07, χ^2^ = 17.05, *df* = 1, *p* < 0.001). Additionally, the Targeted-Support Group reported significantly higher football participation than the High-Need Group (χ^2^ = 5.17, *df* = 1, *p* = 0.023).

These findings indicate that adolescents with different friendship quality profiles exhibit distinct levels of football engagement. The High-Need Group demonstrated the lowest participation, followed by the Targeted-Support Group, while the Peer Leaders Group showed the highest level of engagement. All pairwise comparisons reached statistical significance, suggesting that each profile represents a meaningfully distinct level of football participation.

The overall difference across profiles was significant, Wald χ^2^(2) = 17.22, *p* < 0.001, *ω* = 0.20, Cramér’s *V* = 0.14, both indicating small-to-medium effects.

### Gender differences in football participation

Due to violation of the homogeneity of variance assumption (Levene’s test, *p* < 0.001), Welch’s *F-*test was used to examine gender differences in football participation. Results revealed a significant difference between male and female participants [Welch’s *F* (1, 389.49) = 6.92, *p* = 0.009, η^2^ = 0.016]. Male adolescents reported higher engagement levels (*M* = −0.002, *SD* = 0.80) compared to their female counterparts (*M* = −0.18, *SD* = 0.59).

## Discussion

This study employed Latent Profile Analysis (LPA) to identify distinct sport friendship quality profiles among Chinese adolescent football participants and examined their relationships with football participation. Using the Sport Friendship Quality Scale (SFQS), we identified three meaningful profiles: High-Need Group (16.6%), Peer Leaders Group (53.8%), and Targeted-Support Group (29.6%). These profiles demonstrated significant differences in football engagement, with the High-Need Group showing the lowest engagement and the Peer Leaders Group the highest. These findings advance understanding of peer relationships in youth sport by revealing that adolescents experience friendship quality in qualitatively different ways, and that these differences translate into meaningful variation in participation levels.

The High-Need Group, comprising 16.6% of the sample, displayed consistently low scores across all SFQS dimensions, particularly in Sport Supportiveness and Self-Esteem Enhancement. This pattern suggests that these adolescents face pervasive difficulties in forming and maintaining positive peer connections within the football context. They may struggle with team communication, experience limited emotional support from teammates, and lack the reciprocal encouragement that characterizes high-quality sport friendships (Weiss and Smith, 1999). Previous research has linked weak social ties in sports to increased dropout risk (Pluta et al., 2020), and our BCH results confirm that this group reported the lowest football participation, significantly lower than both the Targeted-Support and Peer Leaders Groups. The comprehensive nature of their social deficits suggests that foundational social skills represent a necessary starting point for interventions targeting this group (Chen et al., 2017; Lin et al., 2024).

In contrast, the Peer Leaders Group, representing the majority of participants (53.8%), demonstrated uniformly high scores across all friendship dimensions, with particular strengths in Positive Character Traits and Self-Esteem Enhancement. These adolescents appear to possess well-developed social competencies that enable them to both give and receive support effectively within team settings, and they likely serve as natural leaders who positively influence team climate (Moran and Weiss, 2006). Interestingly, despite their superior friendship quality, this group showed only moderate levels of football participation, albeit the highest among the three profiles. This finding indicates that positive peer relationships, while beneficial, are not sufficient to guarantee high engagement. Other factors may interact with social dynamics to shape involvement, including intrinsic motivation, perceived competence, enjoyment of the sport itself, and external constraints such as academic pressures (Kirby et al., 2011; Stuntz and Weiss, 2003). For socially competent youth, enhancing friendship quality may be less critical than addressing other factors, such as leadership opportunities or logistical constraints (Bokhorst et al., 2010; Christensen et al., 2024; Fathirezaie et al., 2024).

The Targeted-Support Group (29.6%) displayed a more uneven profile, with moderate scores across most dimensions but relatively lower Sport Supportiveness. While these adolescents demonstrate reasonable competence in areas such as Conflict Resolution and Positive Character Traits, their relative weakness in providing and receiving encouragement during sports activities suggests targeted social needs (Weiss and Smith, 2002). The BCH analysis revealed that this group’s participation fell between the High-Need and Peer Leaders Groups, with all pairwise comparisons reaching statistical significance (Asparouhov and Muthén, 2014). The significant difference between the Targeted-Support and High-Need Groups underscores the value of person-centered methods in detecting subtle but meaningful heterogeneity (Howard and Hoffman, 2018). It also suggests that even partial social competencies are associated with higher engagement than comprehensive deficits, supporting the notion of a social competence threshold (Bengtsson et al., 2024). Once adolescents achieve a basic level of social functioning, further improvements in friendship quality may yield diminishing returns, and other factors become more important determinants of participation. For the Targeted-Support Group, interventions that address their specific weaknesses may help balance their social development.

We further examined whether gender was associated with profile membership by including gender as a covariate in the LPA model. Results indicated that male and female adolescents were similarly distributed across the three friendship quality profiles. This finding indicates that the observed gender differences in football participation, with males reporting higher engagement than females, reflect differences within profiles rather than differential representation across profiles. In other words, boys and girls with comparable friendship quality profiles still exhibit divergent participation levels. This interpretation aligns with research emphasizing sociocultural and structural barriers that disproportionately affect girls’ sports involvement, such as gender stereotypes, unequal access to facilities, and lack of female role models (Boiché et al., 2014; Chalabaev et al., 2013; Gentile et al., 2018). Within each profile, girls may face additional obstacles that dampen their participation despite having similar peer relationship quality to boys (Mousavi and Soltanifar, 2025; Reichardt et al., 2025). These findings underscore the need for gender-sensitive interventions that address barriers operating independently of social dynamics.

### Practical suggestions

The distinct friendship quality profiles identified in this study offer actionable insights for coaches, physical educators, and youth sport administrators. Interventions should address the specific social needs of each group rather than adopt a one size fits all approach.

For the High-Need Group (16.6%), interventions focused on building foundational social skills may be particularly valuable. Structured activities that facilitate positive peer interaction in low-pressure environments can help these adolescents develop confidence in team settings. Coaches might consider incorporating cooperative tasks that require simple communication and shared goal completion.

For the Peer Leaders Group (53.8%), interventions may shift focus from enhancing friendship quality to leveraging existing social competence. Engaging these adolescents as peer mentors or team captains can create opportunities for them to facilitate inclusive environments while also developing leadership skills. Attention to logistical barriers, such as scheduling or access, may further support their sustained participation.

For the Targeted-Support Group (29.6%), interventions addressing specific relational weaknesses warrant attention. Given their relatively lower scores on sport supportiveness, activities designed to encourage peer encouragement and emotional support among teammates may help balance their social competencies. Team-based exercises involving the provision of positive feedback to one another could effectively serve this purpose.

At the program level, integrating social–emotional learning objectives into football curricula represents a broader strategy. The relatively high proportion of peer leaders in this sample suggests that many team environments successfully foster positive peer relationships. Identifying and sharing the practices of such programs could benefit a wider range of adolescents.

Gender differences in participation also warrant targeted attention. Interventions addressing gender-specific barriers, such as promoting inclusive coaching practices and expanding opportunities for girls, may help foster more equitable participation. Schools may consider offering single-gender sessions or involving female coaching role models where feasible.

### Limitations and future directions

Several limitations warrant consideration. Firstly, the cross sectional design prevents causal inferences about how friendship quality affects participation over time. Longitudinal research should examine whether profile membership is associated with future engagement changes. Secondly, self report measures may introduce bias. Future studies could incorporate coach ratings or observational data. Finally, the sample came from one Chinese city, limiting generalizability to other regions or cultures with different social norms around team sports.

Future research could also explore whether gender moderates the relationship between friendship quality and football engagement. Such work would offer deeper insights into how social dynamics influence participation across subgroups. In addition, researchers could design focused interventions for the Targeted Support Group, such as exercises that build sport supportiveness through team bonding activities, to help balance their social development.

## Conclusion

This study identified three distinct sport friendship quality profiles among Chinese adolescent football participants: High-Need, Peer Leaders, and Targeted-Support groups. These profiles differed significantly in football participation, with the High-Need Group demonstrating the lowest engagement and the Peer Leaders Group the highest. The findings highlight that peer relationships play a nuanced role in youth sports participation, operating through different social configurations rather than uniformly across all adolescents. Notably, gender differences in participation emerged within profiles rather than across them, indicating that girls face barriers independent of friendship quality. These results underscore the need for differentiated interventions tailored to specific social needs, particularly for the High-Need Group, alongside gender-sensitive strategies to address structural barriers affecting girls. By revealing how friendship quality patterns combine naturally in real-world populations, this study advances person-centered approaches in sport psychology and provides actionable insights for coaches and educators seeking to foster inclusive, supportive team environments.

## Data Availability

The datasets presented in this study can be found in online repositories. The names of the repository/repositories and accession number(s) can be found in the article/supplementary material.
